# Oral Anticoagulants in the Oldest Old with Recent Stroke and Atrial Fibrillation

**DOI:** 10.1002/ana.26267

**Published:** 2021-11-29

**Authors:** Alexandros A. Polymeris, Kosmas Macha, Maurizio Paciaroni, Duncan Wilson, Masatoshi Koga, Manuel Cappellari, Sabine Schaedelin, Annaelle Zietz, Nils Peters, David J. Seiffge, David Haupenthal, Luise Gassmann, Gian Marco De Marchis, Ruihao Wang, Henrik Gensicke, Svenja Stoll, Sebastian Thilemann, Nikolaos S. Avramiotis, Bruno Bonetti, Georgios Tsivgoulis, Gareth Ambler, Andrea Alberti, Sohei Yoshimura, Martin M. Brown, Masayuki Shiozawa, Gregory Y. H. Lip, Michele Venti, Monica Acciarresi, Kanta Tanaka, Maria Giulia Mosconi, Masahito Takagi, Rolf H. Jäger, Keith Muir, Manabu Inoue, Stefan Schwab, Leo H. Bonati, Philippe A. Lyrer, Kazunori Toyoda, Valeria Caso, David J. Werring, Bernd Kallmünzer, Stefan T. Engelter, NOACISP‐LONGTERM, Erlangen Registry, CROMIS‐2, RAF, RAF‐DOAC, SAMURAI‐NVAF and Verona Registry Collaborators, Stefan T Engelter, Philippe A Lyrer, Leo H Bonati, David J Seiffge, Christopher Traenka, Alexandros A Polymeris, Annaelle Zietz, Nils Peters, Gian Marco De Marchis, Sebastian Thilemann, Nikolaos S Avramiotis, Henrik Gensicke, Lisa Hert, Benjamin Wagner, Fabian Schaub, Louisa Meya, Joachim Fladt, Tolga Dittrich, Urs Fisch, Kosmas Macha, David Haupenthal, Luise Gassmann, Ruihao Wang, Svenja Stoll, Stefan Schwab, Bastian Volbers, Gabriela Siedler, Bernd Kallmünzer, Manuel Cappellari, Bruno Bonetti, Paolo Bovi, Giampaolo Tomelleri, Nicola Micheletti, Cecilia Zivelonghi, Andrea Emiliani, Adrian Parry‐Jones, Chris Patterson, Christopher Price, Abduelbaset Elmarimi, Anthea Parry, Arumug Nallasivam, Azlisham Mohd Nor, Bernard Esis, David Bruce, Biju Bhaskaran, Christine Roffe, Claire Cullen, Clare Holmes, David Cohen, David Hargroves, David Mangion, Dinesh Chadha, Djamil Vahidassr, Dulka Manawadu, Elio Giallombardo, Elizabeth Warburton, Enrico Flossman, Gunaratam Gunathilagan, Harald Proschel, Hedley Emsley, Ijaz Anwar, Ilse Burger, James Okwera, Janet Putterill, Janice O’Connell, John Bamford, John Corrigan, Jon Scott, Jonathan Birns, Karen Kee, Kari Saastamoinen, Kath Pasco, Krishna Dani, Lakshmanan Sekaran, Lillian Choy, Liz Iveson, Maam Mamun, Mahmud Sajid, Martin Cooper, Matthew Burn, Matthew Smith, Michael Power, Michelle Davis, Nigel Smyth, Roland Veltkamp, Pankaj Sharma, Paul Guyler, Paul O’Mahony, Peter Wilkinson, Prabel Datta, Prasanna Aghoram, Rachel Marsh, Robert Luder, Sanjeevikumar Meenakishundaram, Santhosh Subramonian, Simon Leach, Sissi Ispoglou, Sreeman Andole, Timothy England, Aravindakshan Manoj, Frances Harrington, Habib Rehman, Jane Sword, Julie Staals, Karim Mahawish, Kirsty Harkness, Louise Shaw, Michael McCormich, Nikola Sprigg, Syed Mansoor, Vinodh Krishnamurthy, Michela Giustozzi, Monica Acciarresi, Giancarlo Agnelli, Valeria Caso, Cecilia Becattini, Andrea Alberti, Michele Venti, Cataldo D’Amore, Maria Giulia Mosconi, Ludovica Anna Cimini, Maurizio Paciaroni, Fabio Bandini, Georgios Tsivgoulis, Chrissoula Liantinioti, Maria Chondrogianni, Shadi Yaghi, Karen L. Furie, Prasanna Tadi, Marialuisa Zedde, Azmil H Abdul‐Rahim, Kennedy R Lees, Paolo Bovi, Monica Carletti, Alberto Rigatelli, Manuel Cappellari, Jukka Putaala, Liisa Tomppo, Turgut Tatlisumak, Simona Marcheselli, Alessandro Pezzini, Loris Poli, Alessandro Padovani, Vieri Vannucchi, Luca Masotti, Sung‐Il Sohn, Gianni Lorenzini, Rossana Tassi, Francesca Guideri, Maurizio Acampa, Giuseppe Martini, George Ntaios, George Athanasakis, Konstantinos Makaritsis, Efstathia Karagkiozi, Konstantinos Vadikolias, Nicola Mumoli, Franco Galati, Simona Sacco, Cindy Tiseo, Francesco Corea, Walter Ageno, Marta Bellesini, Giovanna Colombo, Giorgio Silvestrelli, Alfonso Ciccone, Alessia Lanari, Umberto Scoditti, Licia Denti, Michelangelo Mancuso, Miriam Maccarrone, Leonardo Ulivi, Giovanni Orlandi, Nicola Giannini, Tiziana Tassinari, Maria Luisa De Lodovici, Christina Rueckert, Antonio Baldi, Danilo Toni, Federica Letteri, Alessio Pieroni, Martina Giuntini, Enrico Maria Lotti, Yuriy Flomin, Odysseas Kargiotis, Theodore Karapanayiotides, Serena Monaco, Mario Maimone Baronello, Laszló Csiba, Lilla Szabó, Alberto Chiti, Elisa Giorli, Massimo Del Sette, Davide Imberti, Dorjan Zabzuni, Boris Doronin, Vera Volodina, Patrik Michel, Peter Vanacker, Kristian Barlinn, Lars‐Peder Pallesen, Jessica Barlinn, Dirk Deleu, Gayane Melikyan, Faisal Ibrahim, Naveed Akhtar, Vanessa Gourbali, Kenichi Todo, Kazumi Kimura, Kensaku Shibazaki, Yoshiki Yagita, Eisuke Furui, Ryo Itabashi, Tadashi Terasaki, Yoshiaki Shiokawa, Teruyuki Hirano, Rieko Suzuki, Kenji Kamiyama, Jyoji Nakagawara, Shunya Takizawa, Kazunari Homma, Satoshi Okuda, Yasushi Okada, Koichiro Maeda, Tomoaki Kameda, Kazuomi Kario, Yoshinari Nagakane, Yasuhiro Hasegawa, Hisanao Akiyama, Satoshi Shibuya, Hiroshi Mochizuki, Yasuhiro Ito, Takahiro Nakashima, Hideki Matsuoka, Kazuhiro Takamatsu, Kazutoshi Nishiyama, Kanta Tanaka, Kaoru Endo, Tetsuya Miyagi, Masato Osaki, Junpei Kobayashi, Takuya Okata, Eijiro Tanaka, Yuki Sakamoto, Keisuke Tokunaga, Hotake Takizawa, Junji Takasugi, Soichiro Matsubara, Kyoko Higashida, Takayuki Matsuki, Naoto Kinoshita, Masayuki Shiozawa, Toshihiro Ide, Takeshi Yoshimoto, Daisuke Ando, Kyohei Fujita, Masaya Kumamoto, Teppei Kamimura, Muneaki Kikuno, Tadataka Mizoguchi, Takeo Sato

**Affiliations:** ^1^ Department of Neurology and Stroke Center University Hospital Basel and University of Basel Basel Switzerland; ^2^ Neurology and Neurorehabilitation, University Hospital for Geriatric Medicine Felix Platter University of Basel Basel Switzerland; ^3^ Department of Neurology University of Erlangen‐Nuremberg Erlangen Germany; ^4^ Neurology – Stroke Unit IRCCS MultiMedica Milan Italy; ^5^ Stroke Research Center, Department of Brain Repair and Rehabilitation UCL Institute of Neurology and The National Hospital for Neurology and Neurosurgery London UK; ^6^ New Zealand Brain Research Institute Christchurch New Zealand; ^7^ Department of Cerebrovascular Medicine National Cerebral and Cardiovascular Center Suita Japan; ^8^ Stroke Unit – Department of Neuroscience Azienda Ospedaliera Universitaria Integrata Verona Italy; ^9^ Clinical Trial Unit, Department of Clinical Research University Hospital Basel and University of Basel Basel Switzerland; ^10^ Stroke Center, Klinik Hirslanden Zurich Switzerland; ^11^ Department of Neurology, Inselspital University Hospital Bern University of Bern Bern Switzerland; ^12^ Second Department of Neurology Attikon University Hospital, National & Kapodistrian University of Athens Athens Greece; ^13^ Department of Neurology University of Tennessee Health Science Center Memphis TN USA; ^14^ Department of Statistical Science University College London London UK; ^15^ Stroke Unit and Division of Cardiovascular Medicine University of Perugia Perugia Italy; ^16^ Liverpool Centre for Cardiovascular Science University of Liverpool, Liverpool Heart and Chest Hospital Liverpool UK; ^17^ Aalborg Thrombosis Research Unit, Department of Clinical Medicine Aalborg University Aalborg Denmark; ^18^ Lysholm Department of Neuroradiology and the Neuroradiological Academic Unit, Department of Brain Repair and Rehabilitation UCL Institute of Neurology London UK; ^19^ Institute of Neuroscience & Psychology University of Glasgow and Queen Elizabeth University Hospital Glasgow UK

## Abstract

**Objective:**

To investigate the safety and effectiveness of direct oral anticoagulants (DOAC) versus vitamin K antagonists (VKA) after recent stroke in patients with atrial fibrillation (AF) aged ≥85 years.

**Methods:**

Individual patient data analysis from seven prospective stroke cohorts. We compared DOAC versus VKA treatment among patients with AF and recent stroke (<3 months) aged ≥85 versus <85 years. Primary outcome was the composite of recurrent stroke, intracranial hemorrhage (ICH) and all‐cause death. We used simple, adjusted, and weighted Cox regression to account for confounders. We calculated the net benefit of DOAC versus VKA by balancing stroke reduction against the weighted ICH risk.

**Results:**

In total, 5,984 of 6,267 (95.5%) patients were eligible for analysis. Of those, 1,380 (23%) were aged ≥85 years and 3,688 (62%) received a DOAC. During 6,874 patient‐years follow‐up, the impact of anticoagulant type (DOAC versus VKA) on the hazard for the composite outcome did not differ between patients aged ≥85 (HR_≥85y_ = 0.65, 95%‐CI [0.52, 0.81]) and < 85 years (HR_<85y_ = 0.79, 95%‐CI [0.66, 0.95]) in simple (p_interaction_ = 0.129), adjusted (p_interaction_ = 0.094) or weighted (p_interaction_ = 0.512) models. Analyses on recurrent stroke, ICH and death separately were consistent with the primary analysis, as were sensitivity analyses using age dichotomized at 90 years and as a continuous variable. DOAC had a similar net clinical benefit in patients aged ≥85 (+1.73 to +2.66) and < 85 years (+1.90 to +3.36 events/100 patient‐years for ICH‐weights 1.5 to 3.1).

**Interpretation:**

The favorable profile of DOAC over VKA in patients with AF and recent stroke was maintained in the oldest old. ANN NEUROL 2022;91:78–88

Atrial fibrillation (AF) becomes more prevalent with increasing age, and both are independent risk factors for ischemic stroke.^1^ As the population ages, the number of patients aged 85 years and older – often termed the *oldest old* – suffering AF‐related ischemic stroke is growing.^2^

In the current guidelines,^3^ direct oral anticoagulants (DOAC) are recommended in patients with AF for recurrent stroke prevention in preference to vitamin K antagonists (VKA) based on the results of the pivotal DOAC randomized controlled trials (RCTs).^4^ However, it is less clear whether this preference can be generalized to include patients: (1) aged ≥85 years, who made up less than 5% of the RCTs population^5^, ^6^, ^7^; or, (2) with recent ischemic stroke, who had been excluded from the RCTs for at least some weeks after stroke.^8^, ^9^

Facing the paucity of randomized evidence, many physicians are reluctant to prescribe DOAC to the oldest old due to assumed safety concerns due to clinical situations particularly prevalent in the oldest old (eg, altered DOAC pharmacokinetics in the presence of unstable or declining renal function, polypharmacy, frailty, malnutrition or reduced body weight),^10^, ^11^ especially for fear of intracranial hemorrhage (ICH).^12^, ^13^ Instead, they may favor VKA,^11^ or withhold oral anticoagulant (OAC) treatment, even in patients who had had an ischemic stroke.^12^ To bridge this evidence gap, systematically ascertained, standardized observational data – known as “real‐world” data – may be useful.^9^, ^14^

With these considerations in mind, we investigated the safety and effectiveness of DOAC compared to VKA in the oldest old with AF and a recent ischemic stroke. In the absence of randomized data, we used prospectively collected, individual patient data pooled within an international collaboration of cohort studies on the use of OAC following ischemic stroke in patients with AF.

## Methods

### Study Design, Patient Population and Data Collection

We used prospectively collected, individual patient data pooled from an established international collaboration of investigator‐initiated cohort studies of patients with AF, recent ischemic stroke or transient ischemic attack (TIA) and OAC treatment, as described previously.^15^ This included 3 single‐center (Basel, Switzerland [NOACISP‐LONGTERM; NCT03826927]^16^; Erlangen, Germany^17^; Verona, Italy^18^) and four multicenter cohorts (CROMIS‐2 [NCT02513316]^19^; RAF^20^; RAF‐DOAC^21^; SAMURAI‐NVAF [NCT01581502]^22^, ^23^). The number of patients contributed by each cohort, as well as the recruitment period and follow‐up duration are summarized in Table S1.

In this study, we included consecutive patients with (1) an index recent (ie, <3 months) ischemic stroke or TIA (as defined previously^15^); (2) nonvalvular AF (either known before index event or first diagnosed thereafter); (3) treatment with DOAC [ie, apixaban, dabigatran, edoxaban, rivaroxaban] or VKA [ie, phenprocoumon, warfarin], initiated within 3 months after the index event; and (4) prospectively ascertained follow‐up data for at least 3 months after the index event for the outcomes recurrent ischemic stroke, ICH and all‐cause death, defined as reported previously.^15^, ^16^ We excluded patients with missing follow‐up or information on age, those with OAC initiation >3 months or unknown, and those with outcome events occurring before OAC initiation.

Data were collected as described in prior research^15^ using standardized forms with predefined variables and pooled in the coordinating center in Basel, Switzerland, where the analysis was performed. We used the following baseline variables: age; sex; National Institutes of Health Stroke Scale (NIHSS) score at baseline; dichotomized type of OAC after index event (DOAC or VKA); time to OAC initiation; concomitant antiplatelet use; history of ischemic stroke or TIA before the index event; history of ICH; diabetes mellitus; hypertension or dyslipidemia; the CHA_2_DS_2_‐VASc score (congestive heart failure, hypertension, age 65–74 or ≥ 75 years, diabetes mellitus, IS or TIA, vascular disease, sex)^24^; estimated glomerular filtration rate (eGFR) using the Chronic Kidney Disease Epidemiology Collaboration [CKD‐EPI] equation^25^ and current smoking, as described previously.^15^

Follow‐up data included length of follow‐up and absence or occurrence and timing of any of the following outcome events, which were defined in line with prior research^15^, ^16^: (1) recurrent ischemic stroke (defined as new neurological deficits with a corresponding finding on neuroimaging); (2) ICH (defined as new neurological deficits with detection of intracranial bleeding on neuroimaging); and (3) all‐cause death, defined as every death irrespective of the cause and regardless of whether the cause was known or not.

### Outcomes

The primary outcome was the time to occurrence of the composite of recurrent ischemic stroke, ICH and all‐cause death, in accordance with prior research.^15^, ^16^ Secondary outcomes were the time to occurrence of each of these outcomes separately.

### Statistical Analysis

We stratified patients' characteristics by dichotomized age (≥85 vs. <85 years) and type of OAC (VKA vs. DOAC). We presented categorical data using frequencies and percentages and continuous data using the median and interquartile range (IQR) or mean and standard deviation (SD) as appropriate. We compared categorical variables using the χ^2^‐test and continuous variables using the Mann–Whitney U test or t‐test as appropriate. We calculated the annualized rate of outcome events as the total of observed events divided by patient‐years of follow‐up for each outcome.

As for the main analysis, we modelled time to primary outcome using Cox proportional hazards regression. For this, we analyzed time to first event after OAC initiation, without considering further events. To assess the effect of age on the performance of OACs, we included type of OAC (DOAC vs. VKA), dichotomized age (≥85 vs. <85 years) and an interaction term between these variables as fixed effects in the model. A significant interaction would indicate that the association between type of OAC and the composite outcome is modified by age and therefore differs in the oldest old compared to their younger counterparts. The model included the participating cohort study as a stratum.

We fitted the model three times according to the predefined analysis plan: (i) simple model including type of OAC and dichotomized age, with and without interaction term; (ii) adjusted model taking into account the known prognostic importance of sex, NIHSS at baseline and CHA_2_DS_2_‐VASc score^24^ (without the age and sex components, modified as in prior research^16^); (iii) weighted model, using the stabilized inverse probability of treatment weights (SIPTW).^26^ We constructed comparable treatment groups (DOAC vs. VKA) with regard to the following potentially outcome‐modifying variables, as in previous research^15^, ^16^: sex, NIHSS at baseline, diabetes mellitus, hypertension, dyslipidemia, eGFR, history of prior stroke or TIA, history of ICH, current smoking, concomitant antiplatelet use and cohort study. We calculated the SIPTW using logistic regression and used robust standard errors for the 95% confidence intervals (CI) and p‐values of the weighted analysis. We imputed missing values in the covariables used in the adjusted and weighted models with simple imputation rules (ie, using the median / mean for continuous variables and the mode [most frequent category] for categorical variables), and report the rate of missing values for all variables. For all models we report the model‐based hazard ratio (HR) estimates along with the 95%‐CI and p‐values. We present the composite outcome data stratified to type of OAC and age group in weighted Kaplan–Meier curves using SIPTW (ie., by weighting each observation by its stabilized inverse probability of treatment with DOAC vs. VKA),^27^ for which we show both the crude and weighted numbers at risk.

We performed the following secondary analyses:

(1) We fitted the Cox models (i) – (iii) described above separately for the individual outcomes recurrent ischemic stroke, ICH and death. To account for competing risks, for these analyses we fitted Cox proportional cause‐specific hazards models treating competing outcomes as censored observations.^28^ With this approach, the competing outcomes influence the measure of association for the outcome of interest by removing at risk patient‐years from the risk set over time.

(2) We analyzed the net clinical benefit (NCB) of DOAC over VKA in patients aged ≥85 and < 85 years. We calculated the NCB by subtracting the weighted rate of excess ICH attributable to DOAC from the rate of excess ischemic stroke prevented by DOAC according to the following formula, as in prior research^29^, ^30^, ^31^, ^32^:

$$\mathrm{NCB}=\left({\text{rate of recurrent ischemic stroke}\;}_{\left[\mathrm{VKA}\;\text{group}\right]}\;-{\text{rate of recurrent ischemic stroke}\;}_{\left[\text{DOAC group}\right]}\right)\;–\mathrm{ICH}\;\text{weight}\;x\;\left({\text{rate of}\;\mathrm{ICH}\;}_{\left[\text{DOAC group}\right]}\;-{\text{rate of}\;\mathrm{ICH}\;}_{\left[\mathrm{VKA}\;\text{group}\right]}\right)$$

The ICH weight reflects the more severe clinical impact in terms of death and disability of ICH relative to ischemic stroke, with values ranging from 1.5 to 3.1 according to previously published weights.^29^, ^30^, ^31^ We performed the NCB analyses for the entire range of weights according to previously used methodology.^32^ We corrected the rate of ischemic stroke and ICH for baseline imbalances between DOAC‐ and VKA‐treated patients using SIPTW as described above and report the NCB in events per 100 patient‐years along with 95%‐CI, calculated based on 1,000 bootstrap replications. For the NCB analyses we considered all patients but those with death as first outcome.

As sensitivity analyses, we repeated the main analysis for the primary (composite) outcome using age as:

(1) a categorical variable, dichotomized to ≥90 vs. <90 years. For this, we refitted all Cox models (i) – (iii) as described above.

(2) a continuous variable, using cubic B‐splines to model the non‐linear association between age and log‐hazard for the composite outcome. For this, we fitted the weighted model (iii) described above twice, with and without the interaction OAC type by age, and compared the two models using a likelihood ratio test. We graphically present the predicted rate of the composite outcome by age stratified to OAC type.

Statistical analyses were performed using R version 3.6.2 (2019‐12‐20) (R Core Team, 2019).

We conducted this study in accordance with the STROBE Statement for observational studies.^33^

### Ethics

The NOACISP‐LONGTERM registry and the current analysis of pooled individual patient data were approved by the ethics committee in Basel, Switzerland (EKNZ 2014–027; PB_2016_00662). Patients provided written informed consent for participation in NOACISP‐LONGTERM. The requirement for additional local ethical approval and patient informed consent differed among participating studies and was acquired by the local investigators as necessary. CROMIS‐2 was approved by the National Research Ethics Committee, London Queen Square and patients with capacity gave informed written consent. When patients could not consent, written consent from a proxy was obtained as defined by relevant local legislation. The SAMURAI‐NVAF registry and the current collaboration were approved by the ethics committee in the National Cerebral and Cardiovascular Center (M23‐18‐3 and M29‐077).

## Results

**FIGURE 1:**
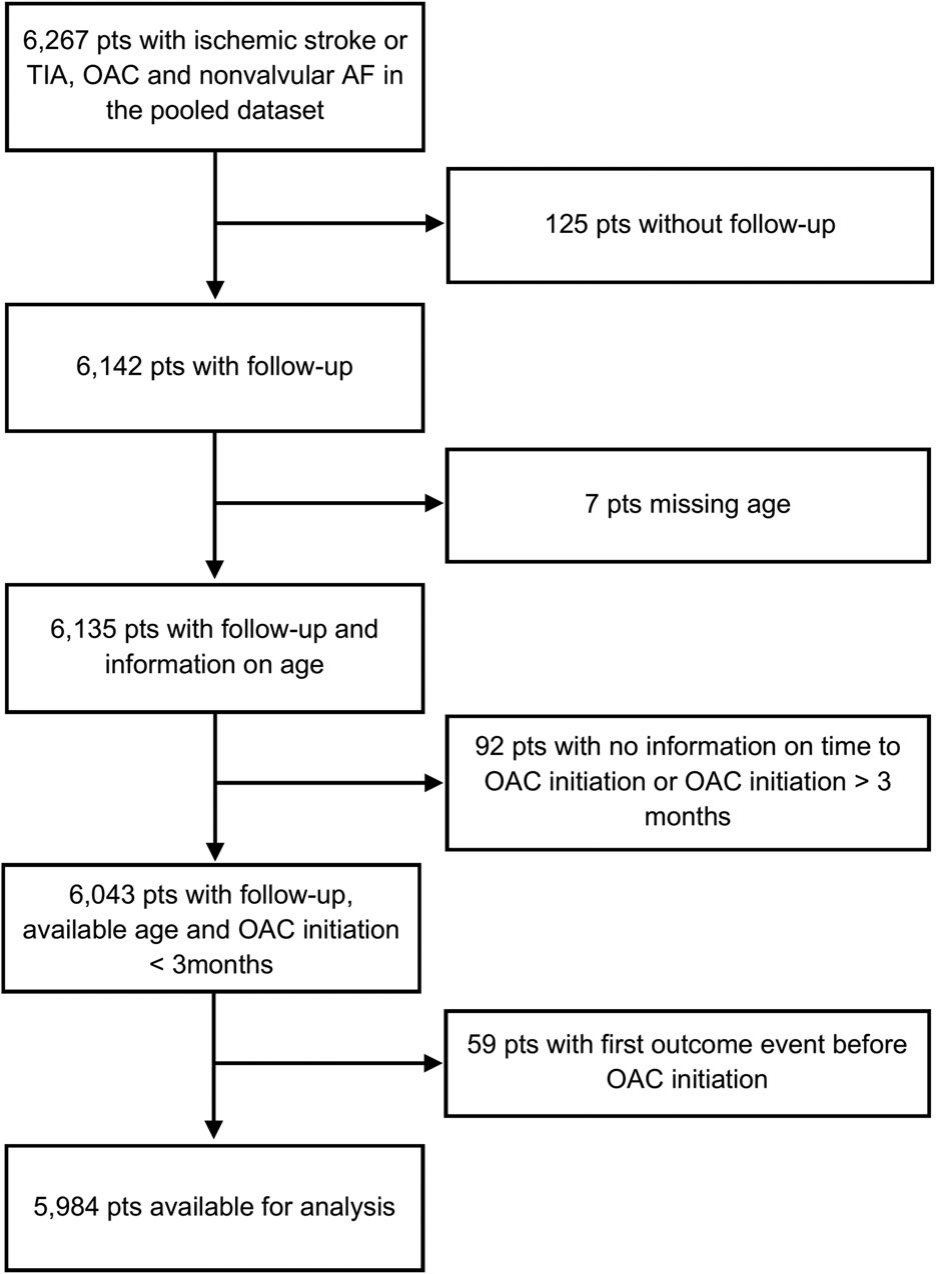
Study Flowchart

In total, 5,984 of 6,267 (95.5%) patients were eligible for analysis. Information on OAC type was complete. Seven patients were excluded for missing age and 125 patients for missing follow‐up data (study flowchart in Fig 1).

### Baseline Characteristics

The index event was ischemic stroke in 5,593 patients (93.5%) and TIA in 391 (6.5%); 2,858 patients (47.8%) were female. The median age was 78 years (IQR 71–84, range 24–102); 1,380 (23.1%) patients were aged ≥85 years and 4,604 (76.9%) were aged <85 years. OAC was initiated at a median (IQR) of 5 (2–11) days after the index event with DOAC in 3,688 patients (61.6%) and VKA in 2,296 patients (38.4%).

#### Patients Aged ≥85 vs. <85 years

The baseline characteristics of the oldest old compared to their younger counterparts are displayed in Table 1. Patients aged ≥85 years were more commonly female and had higher NIHSS scores and lower eGFR, more often hypertension and previous ischemic stroke or TIA, as well as higher CHA_2_DS_2_‐VASc scores compared to younger patients. Diabetes mellitus was more common among younger patients, as was dyslipidemia and current smoking. Time to OAC initiation after index event was shorter in the oldest old.

**TABLE 1:** Patient Characteristics Stratified to Age Group and OAC Type

	Age ≥85 years	Age <85 years		Missing values rate	Age ≥85 years			Age <85 years		
					DOAC	VKA		DOAC	VKA	
Patients, n (%)	1,380 (23.1)	4,604 (76.9)	*p*‐value		865 (62.7)	515 (37.3)	*p*‐value	2,823 (61.3)	1,781 (38.7)	*p*‐value
***Demographics***										
Age, years, median (IQR)	88 (86–90)	75 (69–80)	<0.001	0%	88 (86–90)	88.2 (86–91)	0.001	75.1 (69–80)	75.0 (69–80)	0.119
Female sex, n (%)	881 (63.8)	1,977 (42.9)	<0.001	0%	549 (63.5)	332 (64.5)	0.753	1,249 (44.2)	728 (40.9)	0.027
***Stroke characteristics***										
Ischemic stroke as index event, n (%)	1,292 (93.6)	4,301 (93.4)	0.836	0%	794 (91.8)	498 (96.7)	<0.001	2,599 (92.1)	1,702 (95.6)	<0.001
NIHSS at baseline, median (IQR)	6 (3–12.5)	5 (2–11)	<0.001	7.8%	5 (2–11)	8 (3–16)	<0.001	4 (2–10)	6 (2–12)	<0.001
***Medication details***										
Time to OAC initiation, days, median (IQR)	4 (2–10)	5 (2–11)	0.001	5.1%^a^	4 (2‐9)	4 (2–12)	0.620	5 (2–10)	5 (2–14)	0.081
Concomitant antiplatelet use, n (%)	370 (30.9)	1,255 (30.1)	0.622	10.4%	196 (27.7)	174 (35.6)	0.005	620 (25.4)	635 (36.8)	<0.001
***Risk factors***										
Previous stroke/TIA, n (%)	389 (28.2)	1,110 (24.1)	0.002	0.03%	250 (28.9)	139 (27.0)	0.497	686 (24.3)	424 (23.8)	0.724
Previous ICH, n (%)	17 (1.5)	37 (1.1)	0.322	24.0%	12 (1.7)	5 (1.1)	0.577	17 (0.8)	20 (1.5)	0.078
Diabetes mellitus, n (%)	309 (22.4)	1,200 (26.1)	0.006	0.07%	181 (20.9)	128 (24.9)	0.104	694 (24.6)	506 (28.4)	0.004
Hypertension, n (%)	1,146 (83.3)	3,474 (75.7)	<0.001	0.3%	742 (85.9)	404 (79.1)	0.001	2,169 (77.0)	1,305 (73.6)	0.011
Dyslipidemia, n (%)	521 (43.4)	1,908 (48.7)	0.001	14.5%	365 (51.8)	156 (31.4)	<0.001	1,177 (54.5)	731 (41.7)	<0.001
CHA_2_DS_2_VASc‐Score, mean (SD)	6.0 (1.1)	4.9 (1.6)	<0.001	1.1%	6.0 (1.2)	5.9 (1.1)	0.038	5.0 (1.6)	4.8 (1.6)	<0.001
Modified CHA_2_DS_2_VASc‐Score (without age and sex), mean (SD)	3.3 (1.1)	3.1 (1.2)	<0.001	1.1%	3.4 (1.1)	3.2 (1.0)	0.018	3.2 (1.2)	3.0 (1.3)	<0.001
eGFR, ml/min, mean (SD)	51.0 (24.4)	62.3 (29.3)	<0.001	14.0%	50.4 (25.6)	51.9 (21.9)	0.303	64 (48–83)	66 (51–81)	0.039
Current smoking, n (%)	70 (5.3)	803 (18.0)	<0.001	3.4%	49 (6.0)	21 (4.2)	0.205	501 (18.5)	302 (17.3)	0.331

DOAC = direct oral anticoagulant; eGFR = estimated glomerular filtration rate; ICH = intracranial hemorrhage; NIHSS = National Institutes of Health Stroke Scale; OAC = oral anticoagulant; VKA = Vitamin‐K‐antagonist.

^a^ exact time missing, but all <30 days.

#### Patients with DOAC vs. VKA

There were no substantial differences between DOAC‐ and VKA‐treated patients regarding age (median [IQR] 78 [71–84] years in both groups, *p* = 0.179), sex (48.8% vs. 46.2% female, *p* = 0.055) and time to OAC initiation (median [IQR] 5 [2–10] vs. 5 [2–14] days, *p* = 0.075). Compared to patients with VKA treatment, DOAC‐treated patients had less often ischemic stroke as index event (92.0% vs. 95.8%, *p* < 0.001), reflected in their lower NIHSS scores (median [IQR] 5 [2–10] vs. 6 [2–13], *p* < 0.001). They had less often concomitant antiplatelets (26.0% vs. 36.5%, *p* < 0.001) and diabetes (23.7% vs. 27.6%, *p* = 0.001), but more commonly hypertension (79.1% vs. 74.8%, *p* < 0.001) and dyslipidemia (53.8% vs. 39.4%, *p* < 0.001). DOAC‐treated patients had higher CHA_2_DS_2_‐VASc scores (mean [SD] 5.2 [1.5] vs. 5.0 [1.6], *p* < 0.001). This was largely consistent in the subgroups of patients aged ≥85 vs. <85 years (Table 1).

### Main Analysis – Primary Composite Outcome

During a total follow‐up of 6,874 patient‐years we observed a total of 279 recurrent ischemic strokes, 69 ICH and 737 deaths. This amounted to 994 primary (composite) outcome events, a primary outcome event rate of 14.5%/year. The follow‐up time, number and crude rate of events for the primary outcome stratified to age group and OAC type are given in Table 2.

**TABLE 2:** Follow‐up Time, Number and Crude Rate of Events for the Primary and Secondary Outcomes

		Patient‐years of follow‐up	Number of events (annualized rate)			
			Composite outcome	Recurrent ischemic stroke	Intracranial hemorrhage	All‐cause death
**All**		6,874	994 (14.5%)	279 (4.1%)	69 (1.0%)	737 (10.7%)
**Stratified to age**						
≥85 years		1,502	387 (25.8%)	72 (4.8%)	18 (1.2%)	337 (22.4%)
<85 years		5,372	607 (11.3%)	207 (3.9%)	51 (0.9%)	400 (7.4%)
**Stratified to OAC type**						
DOAC		3,559	491 (13.8%)	150 (4.2%)	26 (0.7%)	351 (9.9%)
VKA		3,316	503 (15.2%)	129 (3.9%)	43 (1.3%)	386 (11.6%)
**Stratified to age and OAC type**						
≥85 years	DOAC	779	181 (23.2%)	40 (5.1%)	8 (1.0%)	152 (19.5%)
	VKA	723	206 (28.5%)	32 (4.4%)	10 (1.4%)	185 (25.6%)
<85 years	DOAC	2,780	310 (11.2%)	110 (4.0%)	18 (0.6%)	199 (7.2%)
	VKA	2,593	297 (11.5%)	97 (3.7%)	33 (1.3%)	201 (7.8%)

DOAC = direct oral anticoagulant; OAC = oral anticoagulant; VKA = Vitamin‐K‐antagonist.

**FIGURE 2:**
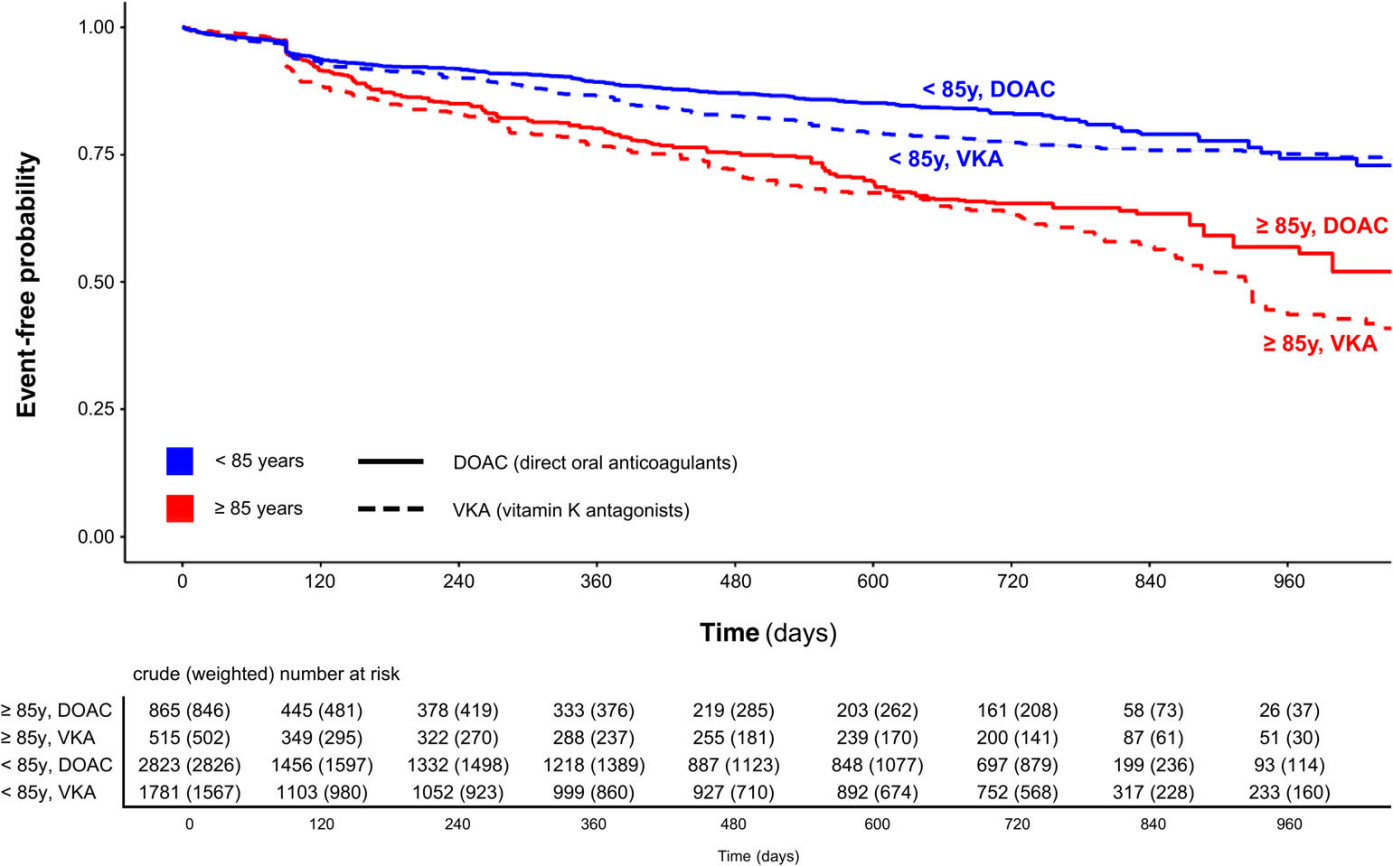
Weighted Kaplan–Meier curves for the composite outcome stratified to anticoagulant type (DOAC / VKA) and age group (≥85 / <85 years)

In the simple Cox model, age < 85 years was associated with a significantly lower hazard for the primary outcome compared to age ≥ 85 years, as indicated by a HR of 0.46 (95%‐CI [0.40, 0.52]). Likewise, DOAC‐treated patients had a lower hazard than VKA‐treated patients with a HR of 0.74 (95%‐CI [0.63, 0.86]). There was no evidence for an interaction between age group and OAC type on their impact on the composite outcome (HR DOAC vs. VKA among patients aged ≥85 years 0.65, 95% CI [0.52, 0.81]; HR DOAC vs. VKA among patients aged <85 years 0.79, 95% CI [0.66, 0.95]; p_interaction_ = 0.129). Consistent findings resulted from repeated analyses refined by adjustment for potential confounders (HR_≥85y_ 0.70, 95%‐CI [0.56, 0.88] and HR_<85y_ 0.87, 95%‐CI [0.73, 1.05]; p_interaction_ = 0.094) and weighting (HR_≥85y_ 0.79, 95%‐CI [0.61, 1.01] and HR_<85y_ 0.88, 95%‐CI [0.71, 1.09]; p_interaction_ = 0.512). Thus, the better performance of DOAC over VKA with regard to the composite outcome was not dependent on age and was maintained in the oldest old. The detailed results of all Cox models for the composite outcome are presented in Table 3. The weighted Kaplan–Meier estimates for the composite outcome stratified to type of OAC and age group are presented in Fig 2.

**TABLE 3:** Cox Models for Time to Composite Outcome

Model (n = 5,984)	Variable	Hazard ratio	95% confidence interval	*p*‐value
simple model	DOAC (vs. VKA)	0.74	[0.63, 0.86]	<0.001
	Age < 85 years (vs. ≥85 years)	0.46	[0.40, 0.52]	<0.001
simple model with interaction term	DOAC (vs. VKA)	0.65	[0.52, 0.81]	<0.001
	Age < 85 years (vs. ≥85 years)	0.41	[0.34, 0.49]	<0.001
	Interaction OAC by age	1.22	[0.94, 1.58]	0.129
adjusted model^a^ with interaction term	DOAC (vs. VKA)	0.70	[0.56, 0.88]	0.002
	Age < 85 years (vs. ≥85 years)	0.42	[0.35, 0.50]	<0.001
	Interaction OAC by age	1.25	[0.96, 1.61]	0.094
weighted model^b^ with interaction term	DOAC (vs. VKA)	0.79	[0.61, 1.01]	0.060
	Age < 85 years (vs. ≥85 years)	0.48	[0.37, 0.61]	<0.001
	Interaction OAC by age	1.12	[0.81, 1.55]	0.512

DOAC = direct oral anticoagulant; OAC = oral anticoagulant; VKA = Vitamin‐K‐antagonist.

^a^ adjustment for sex, National Institutes of Health Stroke Scale score at baseline, modified CHA2DS2‐VASc score (without the age and sex components).

^b^ weighting for sex, National Institutes of Health Stroke Scale score at baseline, history of prior stroke or transient ischemic attack, history of intracranial hemorrhage, diabetes mellitus, hypertension, dyslipidemia, estimated glomerular filtration rate, current smoking, concomitant antiplatelet use, cohort study.

### Secondary Analysis – Individual Outcomes Recurrent Ischemic Stroke, ICH and Death

**FIGURE 3:**
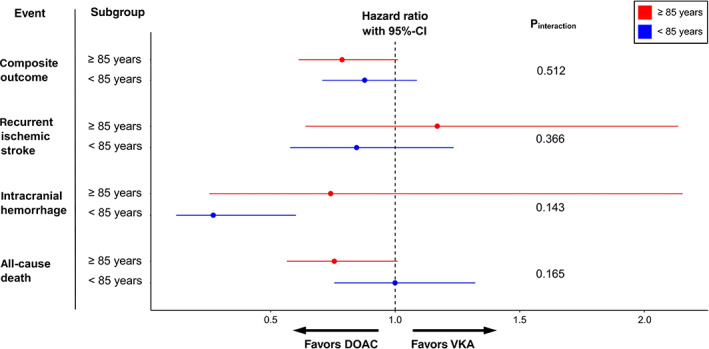
Hazard ratio estimates for the effect of DOAC vs. VKA on the primary composite outcome and all its individual components (accounting for competing risks) stratified to patients aged ≥85 versus <85 years based on the weighted model

Table 2 shows the follow‐up time, number and crude rate of events for the secondary (individual) outcomes in the entire study population and stratified to age group and OAC type. In line with the main analysis of the composite outcome, there was no evidence for an interaction between age and OAC type on the recurrence of ischemic stroke nor the occurrence of ICH in the simple, adjusted and weighted Cox proportional cause‐specific hazards models accounting for competing risks (all p_interaction_ > 0.05). For the outcome death, there was evidence for a weak interaction only in the simple (p_interaction_ = 0.054) and adjusted models (p_interaction_ = 0.029), indicating that the lower hazard for death among patients treated with DOAC as compared to VKA‐treated patients was even more pronounced in the oldest old than in their younger counterparts (simple model: HR_≥85y_ 0.61, 95%‐CI [0.47, 0.79] and HR_<85y_ 0.83, 95%‐CI [0.65, 1.05]; adjusted model: HR_≥85y_ 0.66, 95%‐CI [0.51, 0.86] and HR_<85y_ 0.94, 95%‐CI [0.74, 1.19]). The detailed results of all Cox cause‐specific hazards models for the individual outcomes are presented in Table S2 and Fig 3.

### Secondary Analysis – Net Clinical Benefit

**FIGURE 4:**
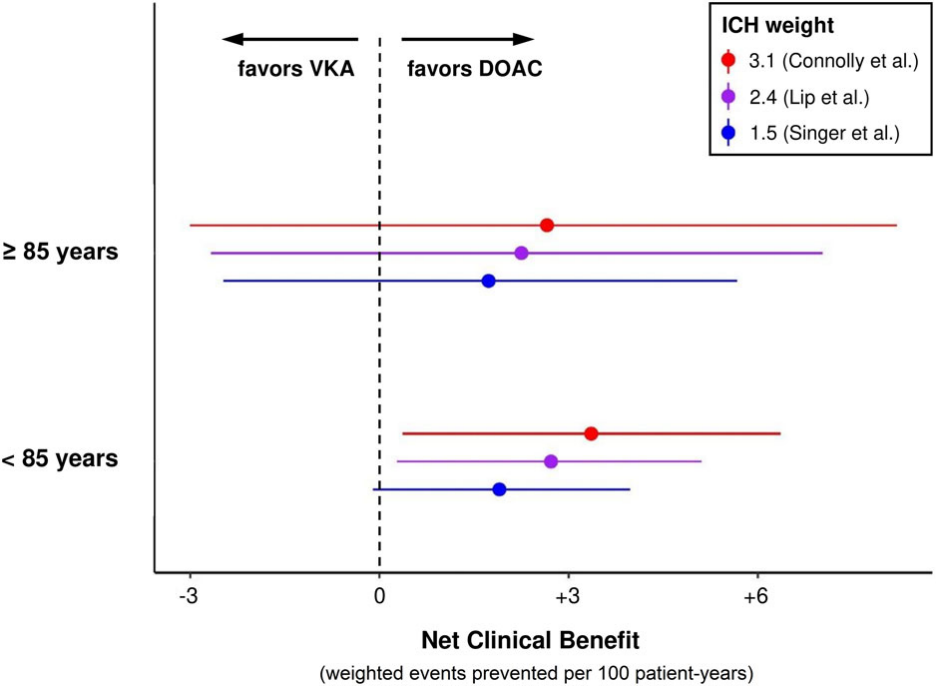
Net clinical benefit of DOAC over VKA with 95% confidence intervals stratified to age group (≥85 / <85 years), using three previously published ICH weights

The point estimates for the NCB of DOAC over VKA were similar in patients aged ≥85 (+1.73 to +2.66) and < 85 years (+1.90 to +3.36 events per 100 patient‐years) and remained positive over the entire range of ICH weights used (1.5 to 3.1), with wide confidence intervals crossing zero in the smaller group of patients aged ≥85 years. Fig 4 depicts the NCB for three previously published ICH weights.^29^, ^30^, ^31^ The detailed results of the NCB analysis are presented in Table S3.

### Sensitivity Analyses – Age Dichotomized at 90 Years and as a Continuous Variable

The sensitivity analyses using age dichotomized to ≥90 (n = 451) vs. <90 years (n = 5,533) showed no evidence for interaction between age and OAC type on the hazard for the composite outcome in the simple (p_interaction_ = 0.283), adjusted (p_interaction_ = 0.514) and weighted (p_interaction_ = 0.433) models, consistent with the main analysis (Table S4).

**FIGURE 5:**
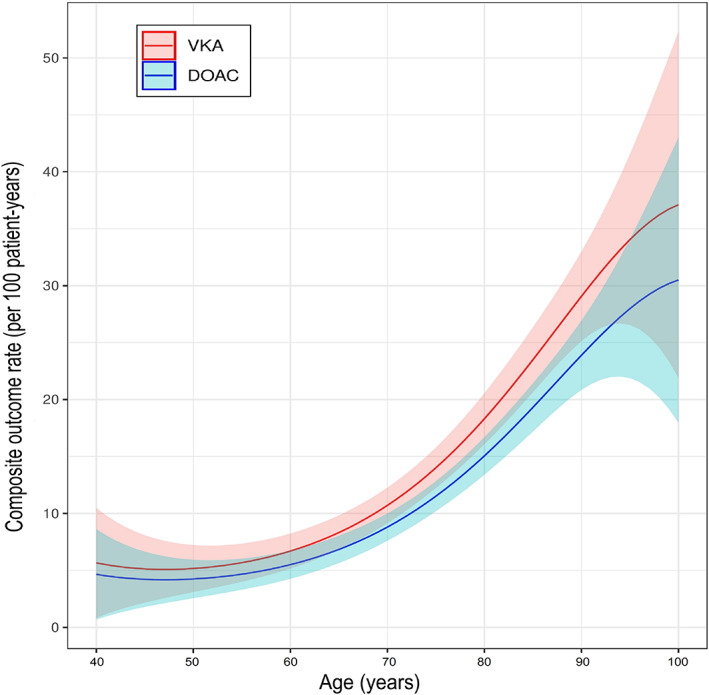
Rate of the composite outcome by age as a continuous variable stratified to type of anticoagulant (DOAC / VKA). The solid lines represent the estimates for each year of age from the weighted model without interaction term and the shaded areas the 95%‐CI.

Using age as a continuous variable, the favorable profile of DOAC over VKA regarding the composite outcome was maintained across the entire age spectrum in the weighted model (Fig 5). There was no evidence that the association between OAC type and composite outcome was modified by age upon comparison of the weighted model with vs. without interaction term (p_likelyhood ratio test_ = 0.623). The hazard for the composite outcome continuously increased with increasing age in a non‐linear fashion.

## Discussion

This study focused on the safety and effectiveness of DOAC versus VKA in the oldest‐old patients with AF and recent stroke or TIA in a real‐world setting. The key finding was that the benefits of DOAC over VKA were consistently maintained in the oldest old, even when potential confounders were accounted for.

In our study, DOAC were associated with a lower hazard than VKA for the composite outcome of recurrent ischemic stroke, ICH and all‐cause death in patients with AF and recent ischemic stroke, independent of age. The favorable profile of DOAC was maintained in the oldest old, whether defined as aged 85 or 90 years or older. This observation is highly relevant for clinical practice as it contradicts the assumptions of many clinicians who are reluctant to use DOAC in this age group, particularly in multimorbid patients.^10^, ^11^ In this context, it is clinically important that the beneficial effect of DOAC over VKA persisted after taking into account the high‐risk profile of the oldest old. Reassuringly, simple, adjusted, as well as weighted models which controlled for the non‐randomized treatment assignment, all yielded consistent results.

Notably, there was no signal of a safety concern regarding ICH risk among the oldest‐old DOAC‐treated patients with recent ischemic stroke, which is a widespread concern.^12^, ^13^, ^34^ In NCB analyses balancing the benefit in stroke reduction against the weighted risk of ICH, the net benefit of DOAC over VKA in these patients was preserved, as indicated by NCB point estimates that were similar in the oldest old as in their younger counterparts, and remained consistently positive across a broad spectrum of ICH weights. Taken together, these findings provide new evidence that the overall beneficial effect of DOAC treatment following recent ischemic stroke is maintained in the oldest old.

These results are clinically important because limited randomized data exist for such patients, as patients with recent stroke within 7 days,^35^ 14 days,^36^, ^37^ or 30 days,^7^ respectively, were excluded from the pivotal DOAC RCTs and the oldest old were severely underrepresented, constituting less than 5% of the RCTs population.^8^, ^9^ While several large observational studies later confirmed the benefits of DOAC in elderly patients with AF,^38^, ^39^, ^40^, ^41^, ^42^, ^43^ they did not examine patients with a recent ischemic stroke. The fact that the elderly in our study had a recent stroke matters, as such patients – compared to those without recent stroke – have a higher risk for hemorrhagic complications, including ICH^44^ and hemorrhagic transformation of the ischemic infarct,^45^ concomitant active small vessel disease^46^, ^47^ and stroke‐induced motor and cognitive deficits with an increased risk of falls.^8^, ^48^

For patients with recent stroke, subgroup analyses in observational studies suggested the safety of DOAC versus VKA for the age groups of ≥75^23^ and > 80 years.^15^, ^49^ As we are not aware of any studies investigating AF patients with both (1) age over 85 years and (2) a recent ischemic stroke, our data address an important evidence gap, mitigating concerns about the applicability of the RCT findings in everyday clinical stroke practice and supporting the current guidelines for prevention of stroke recurrence.^3^

### Strengths and Limitations

Our study has the following strengths: (1) we used individual patient data pooled within an established collaboration of prospective observational studies from Europe and Asia; (2) the high data completeness limits the risk of spurious findings; and (3) the consistency of results both in unadjusted and in adjusted and – most importantly – weighted analyses accounting for potential confounders, as well as in net benefit analyses and in sensitivity analyses focusing on patients ≥90 years or using age as a continuous measure, underlines the robustness of our key finding.

We are aware of the following limitations: (1) as our data are observational rather than randomized, baseline imbalances in the allocation to the type of OAC that were unaccounted for might have introduced bias or confounding; (2) our study included exclusively OAC‐treated patients, so age‐matched stroke patients without OAC treatment were not available for comparison. Of note, the placebo‐controlled ELDERCARE‐AF trial suggested the benefit of anticoagulation even in very elderly patients with AF who were not appropriate candidates for standard anticoagulant treatment^10^; (3) we did not consider extracranial bleeding or myocardial infarction in our analyses, as these outcomes were not available in all participating cohorts; (4) the follow‐up time in the participating cohorts differed, with some reporting over 2 years of follow‐up data, while others were limited to 3 months; (5) our study did not include information on adherence to oral anticoagulants, which was not systematically assessed in most cohorts, although our previous work from the single‐center NOACISP‐LONGTERM cohort indicated high rates of self‐reported adherence both in VKA‐ and DOAC‐treated patients also among the oldest old^50^; (6) Dementia was not an explicit exclusion criterion in any of the contributing cohorts. However, as our study lacked information on the frequency of dementia, it remains unclear whether our key findings are applicable to demented patients, too.

In conclusion, our study provides new and compelling evidence indicating that the benefits of DOAC over VKA in patients with AF and recent stroke are maintained among the oldest old.

## Author Contributions

All authors contributed to study design, data acquisition and analysis, and critically revised the manuscript.

## Potential Conflicts of Interest

MP: speaker honoraria from Sanofi‐Aventis, Boehringer‐Ingelheim, Bayer, BMS, Daiichi‐Sankyo, Pfizer (all manufacturers of anticoagulants). MK: speaker honoraria from Bayer, Nippon Boehringer‐Ingelheim, Daiichi‐Sankyo. MC: consulting fees from Boehringer‐Ingelheim, Pfizer/BMS; advisory board Daiichi‐Sankyo. GMDM: consultant/speaker honoraria from Bayer, travel honoraria from Pfizer. ST: travel grants from BMS/Pfizer. GYHL: Consultant and speaker for BMS/Pfizer, Boehringer‐Ingelheim and Daiichi‐Sankyo; no fees are received personally. LHB: consultancy or advisory board fees or speaker's honoraria from Bayer and BMS. PAL: research grants from Bayer, travel grants from Bayer, Pfizer, advisory board compensation from Bayer, Pfizer, Daiichi‐Sankyo, BMS. KT: lecture honoraria (modest) from Daiichi‐Sankyo, Bayer Yakuhin, Nippon Boehringer‐Ingelheim, BMS. DJW: personal fees from Bayer, Portola (manufacturer of the anticoagulant reversal agent andexanet alfa). STE: research support from Pfizer, Daiichi‐Sankyo; compensation from Stago (manufacturer of coagulation testing systems) for educational material; travel/speaker honoraria from Bayer, Boehringer‐Ingelheim, BMS, Daiichi‐Sankyo; advisory board Bayer, Boehringer‐Ingelheim, BMS. The remaining authors declare no relevant conflicts.

## Supporting information


**Table S1** Participating cohort studies
**Table S2**. Cox proportional cause‐specific hazards models for time to recurrent ischemic stroke, intracranial hemorrhage and death, accounting for competing risks
**Table S3**. Net clinical benefit of DOAC over VKA
**Table S4**. Cox models for time to composite outcome using age dichotomized at 90 years
**Table S5**. Complete list of investigators of the cohort studies that contributed patients to this analysis
