# Supplementary material for: Oral Anticoagulants in the Oldest Old with Recent Stroke and Atrial Fibrillation
Source: Ann Neurol. 2021 Nov 29;91(1):78–88. doi: 10.1002/ana.26267 (PMC9300111; doi:10.1002/ana.26267)
Supplement: Supplementary file 1 — Table S1 Participating cohort studies Table S2. Cox proportional cause‐specific hazards models for time to recurrent ischemic stroke, intracranial hemorrhage and death, accounting for competing risks Table S3. Net clinical benefit of DOAC over VKA Table S4. Cox models for time to composite outcome using age dichotomized at 90 years Table S5. Complete list of investigators of the cohort studies that contributed patients to this analysis [file ANA-91-78-s001.pdf]

Supplementary Material

Oral anticoagulants in the oldest old with recent stroke and atrial fibrillation

Polymeris et al

Table of Contents

Supplementary Table 1. Participating cohort studies 1

Supplementary Table 2. Cox proportional cause-specific hazards models for time to recurrent ischemic stroke, intracranial hemorrhage and death, accounting for competing risks 2

Supplementary Table 3. Net clinical benefit of DOAC over VKA 3

Supplementary Table 4. Cox models for time to composite outcome using age dichotomized at 90 years 4

Supplementary Table 5. Complete list of investigators of the cohort studies that contributed patients to this analysis 5

Supplementary Table 1. Participating cohort studies

Cohort study	Study period	Number of patients contributed to the final pooled cohort in this study	Median follow-up (days)
**Single-center**			
NOACISP-LONGTERM*	2012 – 2019	798	717
Erlangen Registry*	2011 – 2013<br>2016 – 2019	1,174	107
Verona Registry	2013 – 2015	222	90
**Multicenter**			
CROMIS-2	2011 – 2015	1,245	786
RAF	2012 – 2014	553	90
RAF-DOAC	2014 – 2016	868	90
SAMURAI-NVAF	2011 – 2014	1,124	717

* The datasets of these ongoing studies were updated since the initial data pooling (Seiffge et al. Ann Neurol 2019) to include additional patients with index event up to December 31st, 2019.

Supplementary Table 2. Cox proportional cause-specific hazards models for time to recurrent ischemic stroke, intracranial hemorrhage and death, accounting for competing risks

**Model** (N = 5,984)	**Variable**	**Recurrent ischemic stroke**			**Intracranial hemorrhage**			**All-cause death**		
		**Hazard ratio**	**95 % confidence interval**	**p-value**	**Hazard ratio**	**95 % confidence interval**	**p-value**	**Hazard ratio**	**95 % confidence interval**	**p-value**
simple model	DOAC (vs. VKA)	0.90	[0.67, 1.20]	0.463	0.38	[0.20, 0.72]	0.003	0.72	[0.60, 0.88]	<0.001
	Age <85 years (vs. ≥85 years)	0.83	[0.63, 1.10]	0.193	0.78	[0.45, 1.37]	0.391	0.34	[0.29, 0.39]	<0.001
simple model with interaction term	DOAC (vs. VKA)	0.87	[0.53, 1.44]	0.591	0.60	[0.22, 1.69]	0.334	0.61	[0.47, 0.79]	<0.001
	Age <85 years (vs. ≥85 years)	0.82	[0.54, 1.22]	0.323	1.01	[0.48, 2.12]	0.988	0.29	[0.23, 0.36]	<0.001
	Interaction OAC by age	1.04	[0.60, 1.81]	0.885	0.53	[0.17, 1.66]	0.277	1.36	[1.00, 1.86]	0.054
adjusted model* with interaction term	DOAC (vs. VKA)	0.90	[0.55, 1.50]	0.692	0.58	[0.21, 1.63]	0.303	0.66	[0.51, 0.86]	0.002
	Age <85 years (vs. ≥85 years)	0.85	[0.57, 1.29]	0.449	0.91	[0.43, 1.95]	0.814	0.29	[0.23, 0.36]	<0.001
	Interaction OAC by age	1.06	[0.61, 1.85]	0.825	0.55	[0.18, 1.71]	0.300	1.42	[1.04, 1.94]	0.029
weighted model† with interaction term	DOAC (vs. VKA)	1.17	[0.64, 2.14]	0.612	0.74	[0.26, 2.15]	0.581	0.76	[0.57, 1.01]	0.058
	Age <85 years (vs. ≥85 years)	1.06	[0.59, 1.91]	0.835	1.41	[0.53, 3.74]	0.494	0.32	[0.24, 0.43]	<0.001
	Interaction OAC by age	0.72	[0.36, 1.46]	0.366	0.36	[0.09, 1.41]	0.143	1.32	[0.89, 1.96]	0.165

*adjustment for sex, National Institutes of Health Stroke Scale score at baseline, modified CHA_2DS_2-VASc score (without the age and sex components)
†weighting for sex, National Institutes of Health Stroke Scale score at baseline, history of prior stroke or transient ischemic attack, history of intracranial hemorrhage, diabetes mellitus, hypertension, dyslipidemia, estimated glomerular filtration rate, current smoking, concomitant antiplatelet use, cohort study

OAC, oral anticoagulant; DOAC, direct oral anticoagulant; VKA, Vitamin K antagonist

Supplementary Table 3. Net clinical benefit of DOAC over VKA

ICH weight	Patients aged ≥85 years			Patients aged <85 years		
	**weighted rate of events* in VKA-treated patients** [95%-CI] (per 100 patient-years)	**weighted rate of events* in DOAC-treated patients** [95%-CI] (per 100 patient-years)	**NCB of DOAC over VKA** [95%-CI] (per 100 patient-years)	**weighted rate of events* in VKA-treated patients** [95%-CI] (per 100 patient-years)	**weighted rate of events* in DOAC-treated patients** [95%-CI] (per 100 patient-years)	**NCB of DOAC over VKA** [95%-CI] (per 100 patient-years)
**1.5**	8.17 [4.76, 11.51]	6.44 [4.40, 8.60]	+1.73 [-2.47, +5.68]	6.84 [5.13, 8.64]	4.94 [3.93, 5.94]	+1.90 [-0.10, +3.98]
**1.6**	8.33 [4.70, 11.85]	6.54 [4.35, 8.78]	+1.79 [-2.32, +5.80]	6.98 [5.13, 8.76]	4.99 [3.98, 6.00]	+1.99 [-0.05, +4.14]
**1.7**	8.49 [4.98, 11.99]	6.64 [4.29, 8.91]	+1.85 [-2.32, +6.29]	7.12 [5.23, 8.96]	5.04 [3.96, 6.03]	+2.08 [+0.01, +4.10]
**1.8**	8.65 [4.99, 12.21]	6.74 [4.45, 9.01]	+1.91 [-2.60, +6.40]	7.25 [5.26, 9.14]	5.08 [4.03, 6.17]	+2.17 [+0.00, +4.28]
**1.9**	8.80 [5.21, 12.55]	6.84 [4.40, 9.13]	+1.96 [-2.65, +6.26]	7.39 [5.58, 9.27]	5.12 [4.06, 6.20]	+2.27 [+0.11, +4.48]
**2.0**	8.95 [5.02, 12.67]	6.93 [4.47, 9.30]	+2.02 [-2.51, +6.27]	7.53 [5.45, 9.55]	5.17 [4.09, 6.31]	+2.36 [+0.21, +4.59]
**2.1**	9.11 [5.02, 12.95]	7.03 [4.50, 9.50]	+2.08 [-2.66, +6.77]	7.67 [5.59, 9.64]	5.22 [4.14, 6.29]	+2.45 [+0.19, +4.82]
**2.2**	9.27 [5.16, 13.18]	7.13 [4.58, 9.58]	+2.14 [-2.62, +6.69]	7.80 [5.67, 9.85]	5.26 [4.08, 6.40]	+2.54 [+0.13, +4.93]
**2.3**	9.42 [5.56, 13.27]	7.23 [4.75, 9.68]	+2.19 [-2.59, +6.92]	7.94 [5.82, 10.15]	5.31 [4.17, 6.44]	+2.63 [+0.34, +5.07]
**2.4**	9.58 [5.54, 13.55]	7.33 [4.66, 9.98]	+2.25 [-2.67, +7.03]	8.07 [5.86, 10.41]	5.35 [4.19, 6.50]	+2.72 [+0.28, +5.11]
**2.5**	9.73 [5.65, 13.73]	7.42 [4.81, 10.00]	+2.31 [-2.84, +7.28]	8.21 [6.01, 10.38]	5.40 [4.27, 6.52]	+2.81 [+0.43, +5.37]
**2.6**	9.89 [5.72, 14.19]	7.52 [4.81, 10.15]	+2.37 [-2.53, +7.47]	8.34 [6.06, 10.74]	5.44 [4.30, 6.54]	+2.90 [+0.38, +5.37]
**2.7**	10.05 [5.64, 14.44]	7.62 [4.83, 10.39]	+2.43 [-2.70, +7.55]	8.48 [6.15, 10.86]	5.49 [4.33, 6.69]	+2.99 [+0.32, +5.64]
**2.8**	10.20 [5.81, 14.43]	7.72 [4.58, 10.55]	+2.48 [-2.71, +7.58]	8.62 [6.17, 11.09]	5.53 [4.32, 6.69]	+3.09 [+0.51, +5.77]
**2.9**	10.36 [6.00, 14.75]	7.82 [4.89, 10.68]	+2.54 [-2.71, +7.68]	8.76 [6.34, 11.23]	5.58 [4.41, 6.77]	+3.18 [+0.52, +5.88]
**3.0**	10.51 [5.76, 15.39]	7.91 [4.84, 10.87]	+2.60 [-2.93, +8.00]	8.89 [6.36, 11.43]	5.62 [4.41, 6.85]	+3.27 [+0.37, +6.12]
**3.1**	10.67 [5.93, 15.36]	8.01 [5.01, 11.00]	+2.66 [-3.01, +8.21]	9.03 [6.39, 11.65]	5.67 [4.48, 6.94]	+3.36 [+0.37, +6.36]

*calculated as [rate of ischemic stroke + (ICH weight x rate of ICH)]

DOAC, direct oral anticoagulant; VKA, Vitamin K antagonist; ICH, intracranial hemorrhage

Supplementary Table 4. Cox models for time to composite outcome using age dichotomized at 90 years

**Model** (N = 5,984)	**Variable**	**Hazard ratio**	**95% confidence interval**	**p-value**
simple model	DOAC (vs. VKA)	0.74	[0.63, 0.86]	<0.001
	Age <90 years (vs. ≥90 years)	0.45	[0.38, 0.56]	<0.001
simple model with interaction term	DOAC (vs. VKA)	0.62	[0.44, 0.88]	0.006
	Age <90 years (vs. ≥90 years)	0.41	[0.33, 0.52]	<0.001
	Interaction OAC by age	1.21	[0.85, 1.73]	0.283
adjusted model* with interaction term	DOAC (vs. VKA)	0.73	[0.52, 1.03]	0.077
	Age <90 years (vs. ≥90 years)	0.47	[0.37, 0.59]	<0.001
	Interaction OAC by age	1.13	[0.79, 1.60]	0.514
weighted model† with interaction term	DOAC (vs. VKA)	0.74	[0.52, 1.07]	0.111
	Age <90 years (vs. ≥90 years)	0.44	[0.33, 0.58]	<0.001
	Interaction OAC by age	1.18	[0.79, 1.76]	0.433

*adjustment for sex, National Institutes of Health Stroke Scale score at baseline, modified CHA_2DS_2-VASc score (without the age and sex components)

†weighting for sex, National Institutes of Health Stroke Scale score at baseline, history of prior stroke or transient ischemic attack, history of intracranial hemorrhage, diabetes mellitus, hypertension, dyslipidemia, estimated glomerular filtration rate, current smoking, concomitant antiplatelet use, cohort study

OAC, oral anticoagulant; DOAC, direct oral anticoagulant; VKA, Vitamin K antagonist

Supplementary Table 5. Complete list of investigators of the cohort studies that contributed patients to this analysis

**Cohort Study / Investigator**	**Affiliation**
**NOACISP-LONGTERM**	
Stefan T Engelter	University Hospital Basel, Switzerland
Philippe A Lyrer	
Leo H Bonati	
David J Seiffge	
Christopher Traenka	
Alexandros A Polymeris	
Annaelle Zietz	
Nils Peters	
Gian Marco De Marchis	
Sebastian Thilemann	
Nikolaos S Avramiotis	
Henrik Gensicke	
Lisa Hert	
Benjamin Wagner	
Fabian Schaub	
Louisa Meya	
Joachim Fladt	
Tolga Dittrich	
Urs Fisch	
**Erlangen Registry**	
Kosmas Macha	University of Erlangen-Nuremberg, Germany
David Haupenthal	
Luise Gassmann	
Ruihao Wang	
Svenja Stoll	
Stefan Schwab	
Bastian Volbers	
Gabriela Siedler	
Bernd Kallmünzer	
**Verona Registry**	
Manuel Cappellari	Azienda Ospedaliera Universitaria Integrata, Verona, Italy
Bruno Bonetti	
Paolo Bovi	
Giampaolo Tomelleri	
Nicola Micheletti	
Cecilia Zivelonghi	
Andrea Emiliani	
**CROMIS-2**	
Adrian Parry-Jones	Salford Royal Hospital, UK
Chris Patterson	Bradford Royal Infirmary, UK
Christopher Price	Northumbria Hospitals NHS Trust, UK
Abduelbaset Elmarimi	Bedford Hospital, UK

Anthea Parry	Hillingdon Hospital, UK
Arumug Nallasivam	Countess of Chester Hospital, UK
Azlisham Mohd Nor	Derriford Hospital, UK
Bernard Esis	County Durham & Darlington Hospital, UK
David Bruce	County Durham & Darlington Hospital, UK
Biju Bhaskaran	Torbay District Hospital, UK
Christine Roffe	University Hospital of North Staffordshire, UK
Claire Cullen	Aintree University Hospital, UK
Clare Holmes	Bristol Royal Infirmary, UK
David Cohen	Northwick Park Hospital, UK
David Hargroves	William Harvey Hospital, UK
David Mangion	Pilgrim Hospital, Boston, UK
Dinesh Chadha	Doncaster Royal Infirmary, UK
Djamil Vahidassr	Antrim Area Hospital, UK
Dulka Manawadu	King's College Hospital, UK
Elio Giallombardo	Winchester Hospital, UK
Elizabeth Warburton	Addenbrooke's Hospital, UK
Enrico Flossman	Royal Berkshire Hospital, UK
Gunaratam Gunathilagan	Queen Elizabeth The Queen Mother Hospital, UK
Harald Proschel	Dorset County Hospital, UK
Hedley Emsley	Royal Preston Hospital, UK
Ijaz Anwar	University Hospital Hartlepool, UK
Ilse Burger	Kent & Canterbury Hospital, UK
James Okwera	Rotherham General Hospital, UK
Janet Putterill	Epsom Hospital, UK
Janice O'Connell	Royal Sunderland Hospital, UK
John Bamford	Leeds General Infirmary, UK
John Corrigan	Weston General Hospital, UK
Jon Scott	South Tyneside NHS Foundation Trust, UK
Jonathan Birns	St Thomas' Hospital, London, UK
Karen Kee	Croydon University Hospital, UK
Kari Saastamoinen	Royal London Hospital, UK
Kath Pasco	Royal Surrey County Hospital, UK
Krishna Dani	Southern General Hospital, UK
Lakshmanan Sekaran	Luton & Dunstable Hospital, UK
Lillian Choy	St George's Hospital, London, UK
Liz Iveson	York Teaching Hospitals NHS Foundation Trust, UK
Maam Mamun	Medway Maritime Hospital, UK
Mahmud Sajid	Chesterfield Royal Hospital, UK
Martin Cooper	Sherwood Forest Hospital, UK
Matthew Burn	Buckinghamshire Healthcare NHS Trust, UK
Matthew Smith	Airedale General Hospital, UK
Michael Power	Ulster Hospital, UK
Michelle Davis	Royal Victoria Hospital, UK
Nigel Smyth	Royal Hampshire County Hospital, UK
Roland Veltkamp	Charing Cross Hospital, London, UK
Pankaj Sharma	Charing Cross Hospital, London, UK
Paul Guyler	Southend Hospital, UK
Paul O'Mahony	St Helier Hospital, UK

Peter Wilkinson	Ashford & St Peter’s Hospital, UK
Prabel Datta	Mid Yorkshire Hospital, UK
Prasanna Aghoram	Darent Valley Hospital, UK
Rachel Marsh	Leicester Royal Infirmary, UK
Robert Luder	North Middlesex University Hospital, UK
Sanjeevikumar Meenakishundaram	Whiston Hospital, UK
Santhosh Subramonian	Peterborough & Stamford Hospital, UK
Simon Leach	Lincoln County Hospital, UK
Sissi Ispoglou	West Sandwell & Birmingham Hospital, UK
Sreeman Andole	Queen’s Hospital, Romford, UK
Timothy England	Derby Hospital, UK
Aravindakshan Manoj	Liverpool & Broadgreen Hospital, UK
Frances Harrington	The Royal Cornwall Hospital, UK
Habib Rehman	Royal Albert Edward Infirmary, UK
Jane Sword	Royal Devon & Exeter Hospital, UK
Julie Staals	University Hospital Maastricht, the Netherlands
Karim Mahawish	Warrington Hospital, UK
Kirsty Harkness	Royal Hallamshire Hospital, Sheffield, UK
Louise Shaw	Royal United Hospital, Bath, UK
Michael McCormich	Southern Trust Healthcare, UK
Nikola Sprigg	Nottingham University Hospital, UK
Syed Mansoor	Princess Alexandra Hospital, UK
Vinodh Krishnamurthy	Barnet & Chase Farm Hospitals, UK
**RAF / RAF-DOAC**	
Michela Giustozzi	University of Perugia, Italy
Monica Acciarresi	
Giancarlo Agnelli	
Valeria Caso	
Cecilia Becattini	
Andrea Alberti	
Michele Venti	
Cataldo D’Amore	
Maria Giulia Mosconi	
Ludovica Anna Cimini	
Maurizio Paciaroni	
Fabio Bandini	Ospedale San Paolo, Savona, Italy
Georgios Tsivgoulis	University Hospital, National & Kapodistrian University of Athens, Athens, Greece
Chrissoula Liantinioti	
Maria Chondrogianni	
Shadi Yaghi	The Warren Alpert Medical School of Brown University, Providence, RI, USA
Karen L. Furie	
Prasanna Tadi	
Marialuisa Zedde	AUSL – IRCCS, Reggio Emilia, Italy
Azmil H Abdul-Rahim	University of Glasgow, Glasgow, United Kingdom
Kennedy R Lees	
Paolo Bovi	AOUI Verona, Italy
Monica Carletti	
Alberto Rigatelli	

Manuel Cappellari	
Jukka Putaala	Helsinki University Central Hospital, Helsinki, Finland
Liisa Tomppo	
Turgut Tatlisumak	
Simona Marcheselli	Instituto Clinico Humanitas, Rozzano, Milano, Italy
Alessandro Pezzini	University of Brescia, Italy
Loris Poli	
Alessandro Padovani	
Vieri Vannucchi	Santa Maria Nuova Hospital, Firenze, Italy
Luca Masotti	
Sung-Il Sohn	Keimyung University, Daegu, South Korea
Gianni Lorenzini	Ospedale Lotti Pontedera, Azienda USL Toscana Nordovest
Rossana Tassi	AOU Senese, Siena, Italy
Francesca Guideri	
Maurizio Acampa	
Giuseppe Martini	
George Ntaios	University of Thessaly, Larissa, Greece
George Athanasakis	
Konstantinos Makaritsis	
Efstathia Karagkiozi	
Konstantinos Vadikolias	University Hospital of Alexandroupolis, Greece
Nicola Mumoli	Ospedale Civile di Livorno, Italy
Franco Galati	Jazzolino Hospital, Vibo Valentia, Italy
Simona Sacco	University of L'Aquila, Avezzano Hospital, Italy
Cindy Tiseo	
Francesco Corea	San Giovanni Battista Hospital, Foligno
Walter Ageno	Insubria University, Varese, Italy
Marta Bellesini	
Giovanna Colombo	
Giorgio Silvestrelli	ASST di Mantova, Mantova, Italy
Alfonso Ciccone	
Alessia Lanari	
Umberto Scoditti	University of Parma, Italy
Licia Denti	University of Parma, Italy
Michelangelo Mancuso	Azienda Ospedaliero-Universitaria, Pisa, Italy
Miriam Maccarrone	
Leonardo Ulivi	
Giovanni Orlandi	
Nicola Giannini	
Tiziana Tassinari	Santa Corona Hospital, Pietra Ligure (Savona), Italy
Maria Luisa De Lodovici	Neurology, Insubria University, Varese, Italy
Christina Rueckert	Oberschwabenklinik, Ravensburg, Germany
Antonio Baldi	Ospedale di Portogruaro, Portogruaro, Venice, Italy
Danilo Toni	Sapienza University of Rome, Italy
Federica Letteri	
Alessio Pieroni	
Martina Giuntini	Ospedale Apuano, Massa Carrara, Italy
Enrico Maria Lotti	Ospedale di Ravenna Azienda USL della Romagna, Italy
Yuriy Flomin	Universal Clinic 'Oberig' Kyiv, Ukraine

Odysseas Kargiotis	Metropolitan Hospital, Piraeus, Greece
Theodore Karapanayiotides	AHEPA University Hospital, Thessaloniki, Greece
Serena Monaco	Ospedale Civico, Palermo, Italy
Mario Maimone Baronello	
Laszló Csiba	University of Debrecen, Hungary
Lilla Szabó	
Alberto Chiti	Sant'Andrea Hospital, La Spezia, Italy
Elisa Giorli	
Massimo Del Sette	
Davide Imberti	Ospedale Civile di Piacenza, Italy
Dorjan Zabzuni	
Boris Doronin	Novosibirsk State Medical University, Russia
Vera Volodina	
Patrik Michel	Hopitalier Universitaire Vaudois, Lausanne, Switzerland
Peter Vanacker	Born Bunge Institute, Antwerp University Hospital, Belgium
Kristian Barlinn	Dresden University Stroke Center, Dresden, Germany
Lars-Peder Pallesen	
Jessica Barlinn	
Dirk Deleu	Hamad Medical Corporation, Doha, Qatar
Gayane Melikyan	
Faisal Ibrahim	
Naveed Akhtar	
Vanessa Gourbali	Evangelismos Hospital, Athens, Greece
**SAMURAI-NVAF**	
Kenichi Todo	Osaka University Graduate School of Medicine, Japan
Kazumi Kimura	Kawasaki Medical School, Japan
Kensaku Shibazaki	
Yoshiki Yagita	
Eisuke Furui	Kohnan Hospital, Japan
Ryo Itabashi	
Tadashi Terasaki	Japanese Red Cross Kumamoto Hospital, Japan
Yoshiaki Shiokawa	Kyorin University School of Medicine, Japan
Teruyuki Hirano	
Rieko Suzuki	
Kenji Kamiyama	Nakamura Memorial Hospital, Japan
Jyoji Nakagawara	
Shunya Takizawa	Tokai University School of Medicine, Japan
Kazunari Homma	
Satoshi Okuda	NHO Nagoya Medical Center, Japan
Yasushi Okada	
Koichiro Maeda	
Tomoaki Kameda	Jichi Medical University School of Medicine, Japan
Kazuomi Kario	
Yoshinari Nagakane	Kyoto Second Red Cross Hospital, Japan
Yasuhiro Hasegawa	St Marianna University School of Medicine, Japan
Hisanao Akiyama	
Satoshi Shibuya	South Miyagi Medical Center, Japan
Hiroshi Mochizuki	

Yasuhiro Ito	TOYOTA Memorial Hospital, Japan
Takahiro Nakashima	NHO Kagoshima Medical Center, Japan
Hideki Matsuoka	
Kazuhiro Takamatsu	Brain Attack Center Ota Memorial Hospital, Japan
Kazutoshi Nishiyama	Kitasato University School of Medicine, Japan
Kanta Tanaka	National Cerebral and Cardiovascular Center, Japan
Kaoru Endo	
Tetsuya Miyagi	
Masato Osaki	
Junpei Kobayashi	
Takuya Okata	
Eijiro Tanaka	
Yuki Sakamoto	
Keisuke Tokunaga	
Hotake Takizawa	
Junji Takasugi	
Soichiro Matsubara	
Kyoko Higashida	
Takayuki Matsuki	
Naoto Kinoshita	
Masayuki Shiozawa	
Toshihiro Ide	
Takeshi Yoshimoto	
Daisuke Ando	
Kyohei Fujita	
Masaya Kumamoto	
Teppei Kamimura	
Muneaki Kikuno	
Tadataka Mizoguchi	
Takeo Sato	
